# Method for semi-automated microscopy of filtration-enriched circulating tumor cells

**DOI:** 10.1186/s12885-016-2461-4

**Published:** 2016-07-14

**Authors:** Emma Pailler, Marianne Oulhen, Fanny Billiot, Alexandre Galland, Nathalie Auger, Vincent Faugeroux, Corinne Laplace-Builhé, Benjamin Besse, Yohann Loriot, Maud Ngo-Camus, Merouan Hemanda, Colin R. Lindsay, Jean-Charles Soria, Philippe Vielh, Françoise Farace

**Affiliations:** “Circulating Tumor Cells” Translational Platform AMMICA CNRS UMS3655–INSERM US23, Gustave Roussy, Université Paris-Saclay, F-94805 Villejuif, France; INSERM U981 “Identification of Molecular Predictors and new Targets for Cancer Treatment”, F-94805 Villejuif, France; Univ Paris Sud, Université Paris-Saclay, F-94270 Le Kremlin-Bicêtre, France; Pathology Imaging, Leica Biosystems, F92737 Nanterre, France; Department of Biopathology, Gustave Roussy, Université Paris-Saclay, Villejuif, France; Imaging and Cytometry Platform AMMICA CNRS UMS3655–INSERM US23, Gustave Roussy, Université Paris-Saclay, Villejuif, France; Department of Medicine, Gustave Roussy, Université Paris-Saclay, Villejuif, France

**Keywords:** Circulating tumor cells, Filtration enrichment, Fluorescent staining, FA-FISH, Predictive biomarkers

## Abstract

**Background:**

Circulating tumor cell (CTC)-filtration methods capture high numbers of CTCs in non-small-cell lung cancer (NSCLC) and metastatic prostate cancer (mPCa) patients, and hold promise as a non-invasive technique for treatment selection and disease monitoring. However filters have drawbacks that make the automation of microscopy challenging. We report the semi-automated microscopy method we developed to analyze filtration-enriched CTCs from NSCLC and mPCa patients.

**Methods:**

Spiked cell lines in normal blood and CTCs were enriched by ISET (isolation by size of epithelial tumor cells). Fluorescent staining was carried out using epithelial (pan-cytokeratins, EpCAM), mesenchymal (vimentin, N-cadherin), leukocyte (CD45) markers and DAPI. Cytomorphological staining was carried out with Mayer-Hemalun or Diff-Quik. *ALK*-, *ROS1*-, *ERG*-rearrangement were detected by filter-adapted-FISH (FA-FISH). Microscopy was carried out using an Ariol scanner.

**Results:**

Two combined assays were developed. The first assay sequentially combined four-color fluorescent staining, scanning, automated selection of CD45^−^ cells, cytomorphological staining, then scanning and analysis of CD45^−^ cell phenotypical and cytomorphological characteristics. CD45^−^ cell selection was based on DAPI and CD45 intensity, and a nuclear area >55 μm^2^. The second assay sequentially combined fluorescent staining, automated selection of CD45^−^ cells, FISH scanning on CD45^−^ cells, then analysis of CD45^−^ cell FISH signals. Specific scanning parameters were developed to deal with the uneven surface of filters and CTC characteristics. Thirty z-stacks spaced 0.6 μm apart were defined as the optimal setting, scanning 82 %, 91 %, and 95 % of CTCs in *ALK*-, *ROS1*-, and *ERG*-rearranged patients respectively. A multi-exposure protocol consisting of three separate exposure times for green and red fluorochromes was optimized to analyze the intensity, size and thickness of FISH signals.

**Conclusions:**

The semi-automated microscopy method reported here increases the feasibility and reliability of filtration-enriched CTC assays and can help progress towards their validation and translation to the clinic.

**Electronic supplementary material:**

The online version of this article (doi:10.1186/s12885-016-2461-4) contains supplementary material, which is available to authorized users.

## Background

Circulating tumor cells (CTCs) captured from blood as a liquid biopsy are currently a very active and promising area in translational cancer research [1]. CTCs are very rare cells – occurring at rates as low as one cell per 10^6^ or 10^7^ leukocytes – which detach from a primary tumor or metastatic site, circulate in the peripheral blood and may colonize secondary sites to form metastasis [2, 3]. To date, identification and characterization of CTCs has been hampered by their rarity, their phenotypical and genetic heterogeneity and the technical limitation of current assays [4]. As a consequence, CTC detection methods commonly rely on two steps, a first enrichment step based on either CTC phenotypical or physical properties, and a secondary step of detection to increase the sensitivity of the assay. The semi-automated CellSearch platform (Janssen Diagnostics, LLC, Raritan, USA) enriches CTC candidates through ferromagnetic beads coated with epithelial cell adhesion molecule (EpCAM) specific antibody and defines CTCs according to the presence of cytokeratins (CK8, CK18 and CK19) and the absence of the common leukocyte antigen CD45 [5]. In prospective multicenter studies in metastatic breast, prostate (mPCa) and colon cancers [6–8], CTC levels using this platform were shown to have prognostic significance, and extensive analytical validation and clinical qualification led the U.S. Food and Drug Administration (FDA) to approve CellSearch as an aid to prognosis in these tumors. In non-small-cell lung cancer (NSCLC), CTC levels were also reported to have a prognostic value, but the number of CTCs caught by the CellSearch remains low in this tumor type even in patients with metastatic advanced disease [9]. The potential reason for this is that CTCs have lost epithelial features, expressing markers of the epithelial-mesenchymal transition (EMT) which can be missed by the CellSearch [10–12]. In addition to prognostic utility, CTCs are currently extensively studied to detect predictive biomarkers and screen eligible patients for targeted therapies [13–20]. Moreover analyses performed on CTCs could be repeated at different time-points during treatment to monitor response and guide therapeutic decisions. Performing molecular analysis in CTCs analyzed by the CellSearch is relatively challenging in cancers such as NSCLC due to the low numbers of CTCs captured and the processing conditions of the system.

The limitations of the CellSearch and other antibody-based capture methods have stimulated the development of other technologies for CTC enrichment and detection [4]. An alternative approach consisting of distinguishing CTCs from normal hematopoietic cells according to size is being actively explored. Several filter devices have been described including ISET (ISET, isolation by size of epithelial tumor cells, Rarecells, Paris, France) [21–23], ScreenCell [24], the Accucyte system [25], CellSieve [26], the Portable Filter-based Microdevice [27], VyCAP [28], the Siemens prototype [29] and the Parsortix system [30]; allowing identification of CTCs according to cytomorphological, phenotypical or molecular characteristics. Ours and other groups have reported that higher numbers of CTCs are captured in cancers such as NSCLC and mPCa using a filtration system compared to CellSearch [10, 26, 31, 32]. CTCs expressing mesenchymal and epithelial markers were reported in NSCLC, mPCa and breast cancer patients using filtration enrichment [11, 12, 26, 33, 34] and CTCs enriched by ISET were reported to have a prognostic value in resected NSCLC patients [31]. By capturing CTCs independent of surface-marker expression, filtration methods may offer a significant advantage for covering the phenotypic and genetic heterogeneity of CTCs, and identification of predictive markers. We and another group reported the detection of *ALK*-rearrangement in CTCs ISET-enriched by filtration in NSCLC patients with an *ALK*-rearranged tumor [17, 18, 35] as well *ROS1*-rearrangement in CTCs from NSCLC patients with an *ROS1*-rearranged tumor [20].

While offering important advantages such as sensitivity of CTC capture and flexibility for CTC characterization following filtration, filtration systems have drawbacks. One problem is that it is difficult to design a filter membrane which sits entirely flat, regardless of the material used: virtually all filter membranes developed today are not microscopically flat. Another is that pores inevitably retain white blood cell debris and fluorescence signals which disturb microscopy analysis, thus cells placed on pores are frequently difficult to analyze. These two problems make the automation of microscopy challenging to implement. Given these difficulties the microscopy analysis of CTCs enriched on filters remains manual, time-consuming due to the high numbers of white blood cells present on filters, and highly operator-dependent. Although automation is an essential step for filtration-enriched CTC assays to progress to the clinic, there is today no published method reporting the semi-automation of microscopy and image analysis of filtration-enriched CTCs.

Here we report the semi-automated method established to analyze filtration-enriched CTCs from NSCLC and mPCa according to two combined assays i.e (i) fluorescent staining and high-resolution cytomorphology; (ii) fluorescent staining and fluorescent *in situ* hybridization (FISH). The first method aims to identify CTCs according to both phenotypical and cytomorphological parameters and includes the establishment of scanning parameters for selecting and creating an image gallery of CD45^−^ cells, and characterizing CTCs. The second relies on the detection of molecular biomarkers by establishing FISH scanning parameters (z-stacking, step i.e. distance between two z-stacks, exposure time) for optimal FISH signal identification in filtration-enriched CTCs.

## Methods

### Patients

NSCLC and mPCa patients were recruited at the Gustave Roussy, Paris, France. Informed written consent for blood sample collection was obtained from all patients (IDRCB2008-A00585-50). The study was approved by local institutional board and ethics committees. Blood was collected into EDTA tubes.

### Blood sample collection and enrichment of CTCs by ISET

CTC enrichment by the ISET filtration system (RareCells, Paris, France) was carried out according to the manufacturer’s protocol, as previously reported [10, 11]. To preserve cell integrity, the filtration pressure was optimized to -7 kPa. After processing, filters were dried, wrapped in an aluminum sheet and stored frozen in plastic bag containing a silica gel desiccant at -20 °C until use.

### Fluorescent staining of filtration-enriched CTCs

ISET filters are composed of 10 spots. Each spot (corresponding to filtration of 1 mL blood) was cut out for independent analysis. Filters were thawed and individual spots were immobilized on glass slides using adhesive ribbon. A ‘snick’ was made on each spot to allow the precise relocation of cells between fluorescent staining and cytomorphological staining. After rehydratation in TBS 1X (Thermo Fisher Scientific Inc., Waltham, MA, USA), cell permeabilization was carried out by incubating filters for 7 min at room temperature in TBS 1X-Triton X-100 0.2 % (Roche, Sigma-Aldrich Co. LLC., Saint-Louis, MO, USA). After a wash with TBS 1X, saturation was carried out by incubating filters for 25 min at room temperature in TBS 1X-normal goat serum 5 % (Thermo Fisher Scientific Inc.). Epithelial markers were employed in the “green” channel including mouse anti-pancytokeratin monoclonal antibodies (clone A45-B/B3, AS Diagnostik, Hueckeswagen, Germany; clone C11, Novus Biological, Littleton, CO, USA; clone KL1, Beckman Coulter, Brea, CA, USA; clone OV-TL 12/30, Dako, Les Ulis, France) directly conjugated to Alexa Fluor (AF) 488 using the Zenon Mouse IgG Labeling Kit (Thermo Fisher Scientific Inc.) and EpCAM/CD326 AF488 (clone VU1D9, Novus Biological). An anti-vimentin (clone V9, Santa Cruz Biotechnology, Heidelberg, Germany) or an anti-N-cadherin (clone 32/N-Cadherin, BD Biosciences, Franklin Lakes, NJ, USA) conjugated in AF546 and allophycocyanin (APC)-conjugated anti-CD45 (clone HI30, BD Biosciences) were used. Antibodies incubation was carried out 25 min in a humidity dark chamber. After two washes with TBS 1X-Tween20 0.05 % (Dako) and TBS 1X, 4′,6-diamidino-2-phenylindole (DAPI) or Hoechst 33342 (Sigma-Aldrich) was added for 10 min. ISET spots were mounted between slide and coverslip using Ibidi mounting medium (Biovalley, Nanterre, France). Slides were stored at +4 °C until scanning.

### Cytomorphological staining of filtration-enriched CTCs

After fluorescence scanning, the coverslip and the mounting medium were removed using a wash of PBS 1X, filters were stained with Mayer Hemalun (RAL Diagnostics, Martillac, France) at room temperature for 30 min or with Diff-Quik (Siemens Healthcare diagn., Munich, Germany) according to the manufacturer's protocol. ISET spots were mounted using Ibidi mounting medium and stored at +4 °C until scanning.

### Scanning and image analysis of combined fluorescent and cytomorphological staining in filtration-enriched CTCs

Scanning and image analysis were carried out using an Ariol scanning system (Leica Biosystems Richmond Inc., Richmond, IL, USA) including a Leica DM6000 B microscope with multibay stages (MB 8). Single interference filter sets for blue (DAPI), green (FITC), red (Texas Red) and dark red (Cy5) filters were used. Calibrations were performed using the Ariol Scan application 4.0.1.5 (Leica Biosystems Richmond Inc.). After delineation of the scanning area (i.e. one entire ISET spot) at ×5 magnification, gain was set at maximum (255) to eliminate risk of fluorochrome bleaching. Exposure time was calibrated for each channel at ×20 magnification. Using only one parameter (i.e. exposure time for adjusting fluorochrome exposure) allowed to compare settings between scans done at different times or by different users. Exposure time for epithelial markers was adjusted to have a very low signal on CD45^+^ cells (at limit of the background noise) while the exposure time of the mesenchymal marker was set to have a saturated signal on CD45^+^ cells which are known to be strongly positive. Offset was calibrated as high as possible in the DAPI channel to individualize at best nucleus. Selection of CD45^−^ from CD45^+^ elements was carried out in real time by the Ariol scanning system. The fluorescence scan takes ~1 h30 for one ISET spot. For cytomorphological staining scanning a pre-scan of the whole spot was performed at ×5 in the brightfield channel, the snick being used to precisely relocate cells. The cytomorphological staining scan takes ~45 min at x 20 magnification. All images were analyzed with the Ariol Review application 4.0.1.5 (Leica Biosystems Richmond Inc.).

### Combination of fluorescent staining and FA-FISH of filtration-enriched CTCs

Filters were thawed and individual spots were immobilized on glass slides using adhesive ribbon. A snick was made on each spot for precise CD45^−^ cell relocation for filter-adapted-FISH (FA-FISH) scanning. After rehydratation in TBS 1X (Dako), filters were incubated for 5 min at 98 °C in TBS 1X-EDTA (Dako) and then 30 min in TBS 1X-normal mouse serum 5 % (Thermo Fisher Scientific Inc.). Fluorescent staining was performed overnight with anti-CD45 in a dark humidity chamber. After three washes with TBS 1X-Tween20 0.05 % followed by TBS 1X, nuclear staining was performed using DAPI for 15 min. For FA-FISH, filters were then washed with TBS 1X-Tween 20 0.05 %, incubated for 30 min at room temperature in methanol:acetic acid (9:1) solution and then digested at 37 °C with a pepsin (Sigma-Aldrich) solution at 10 % in 0.01 N HCL. After washing with PBS 1X, filters were fixed at room temperature in formaldehyde solution (Sigma-Aldrich) and dehydrated in successive baths containing increasing concentrations of ethanol (VWR International, Radnor, PA, USA). FA-FISH experiments were performed using several probes according to the manufacturer’s protocol: the Vysis LSI *ALK* Break Apart Rearrangement Probe Kit (Abbott Molecular Inc., Des Plaines, IL, USA); the Vysis 6q22 *ROS1* Break Apart FISH probe RUO Kit (Abbott Molecular Inc.); the ZytoLight SPEC *RET* Dual Color Break (ZytoVision GmbH, Bremerhaven, Germany); the Vysis *EGFR*/*CEP7* FISH Probe Kit (Abbott Molecular Inc.); the Cytocell *c*-*met* amplification Probe Kit (Cytocell Ltd., Cambridge, UK); the ZytoLight SPEC *FGFR1*/*CEN8* Dual Color Probe (ZytoVision GmbH); the *FIP1L1*-*PDGFRA* FISH DNA Probe (Dako) with the TelVysion 4q (Abbott Molecular Inc.); the Vysis *AR* amplification Probe Kit (Abott Molecular Inc.); the Kreatech *ERG* Break Apart Rearrangement Probe Kit (Kreatech Diagnostics, Amsterdam, Netherlands). After hybridization, filters were washed in stringent wash buffer 1X (Dako) and wash buffer 1X (Dako), then dehydrated again in successive ethanol solutions. Filters were finally mounted using DAPI mounting medium (Dako). All FISH probes were optimized for FA-FISH using cancer cell lines spiked in normal blood and filtered by ISET (Additional file 1: Figure S1).

### Scanning and image analysis of combined fluorescent staining and FA-FISH in filtration-enriched CTCs

Scanning and image analysis of fluorescent staining were carried out as described above. For scanning FA-FISH, a pre-scan of the whole ISET spot was first performed at ×5 magnification in the DAPI channel to precisely relocate cells. Thanks to the snick made on the spot, the fluorescence and the FA-FISH scans were linked. A scan pass in the DAPI channel at ×10 magnification was developed for adjustment of focus and FISH capture was performed at ×63 magnification in the small regions containing CD45^−^ cells. The z-stack number, step, gain, offset and exposure time (ranging from 5 to 100 ms) were calibrated at ×63 magnification. The Ariol scanning system adjusted the focus on DAPI every frame. All images were analyzed with the Ariol Review application 4.0.1.5. To determine the best number of z-stacks, filter enriched-CTCs from patients with *ALK*-, *ROS1*- and *ERG*-rearranged tumors were scanned six times from 5 z-stacks to 30 z-stacks. For the best step determination, filter enriched-CTC were scanned four times with steps ranging from 0.5 to 0.8 μm. No bleaching was observed.

### Cell lines

The NCI-H2228, HCC78, NCI-H661, A549, H1975 and LNCaP cell lines were cultured in RPMI medium 1640 (Thermo Fisher Scientific Inc.) and TPC1, A-431, MB-MDA-134, Res259, VCaP in DMEM medium (Thermo Fisher Scientific Inc.) supplemented with 10 % fetal bovine serum and maintained in a humidified incubator in 5 % CO_2_ at 37 °C. Cell lines were spiked at various dilutions into blood from healthy donors filtered on ISET, as done for patient samples.

## Results

### Semi-automated microscopy method to identify filtration-enriched CTCs by combined phenotypical and cytomorphological analysis

**Fig. 1:**
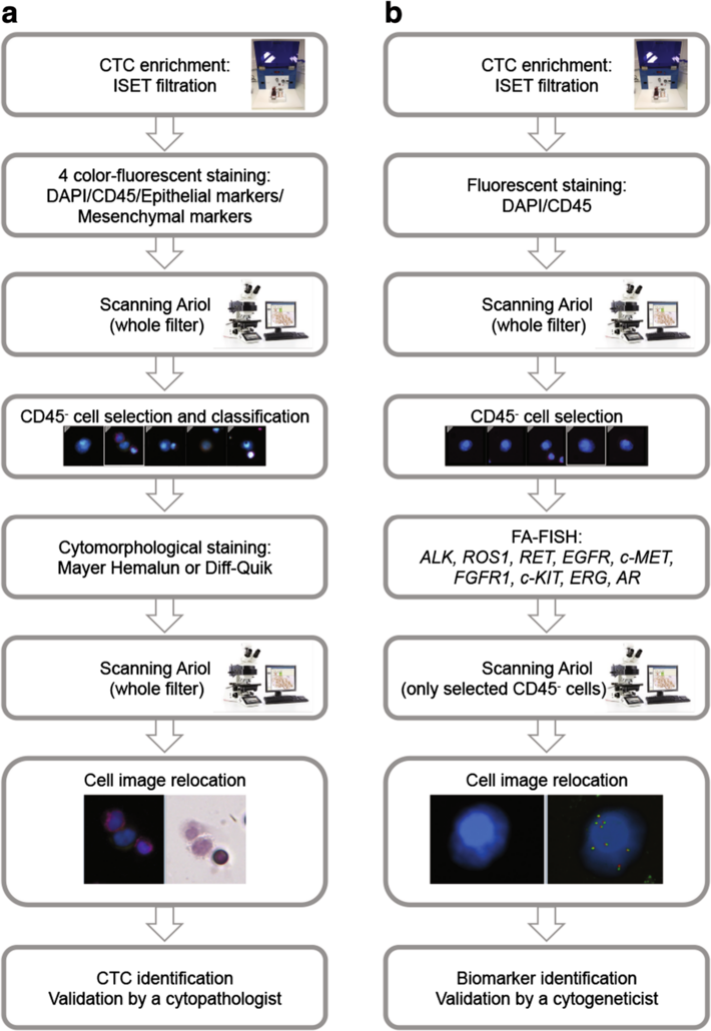
Experimental process for the two combined assays. **a** Schematic workflow for the identification of filtration-enriched CTCs by combined fluorescent staining and high-resolution cytomorphology. **b** Schematic workflow for detection of molecular biomarkers in filtration-enriched CTCs by combined fluorescent staining and fluorescent *in situ* hybridization (FISH)

CTCs are most often identified phenotypically through the detection of epithelial markers by immunofluorescent staining. Filtration-enriched CTCs may also be identified according to true cytomorphological criteria such as nucleus size (compared with the size of the filter pores (8 μm) corresponding to the internal control) and irregularity of the nuclear membrane, the presence of a visible cytoplasm and a high nuclear-to-cytoplasmic ratio [10, 31, 36]. To further characterize filtration-enriched CTCs, we established a method where CTCs were identified by combining phenotypical (immunofluorescent staining) and cytomorphological characterization, and report here the semi-automated scanning and image analysis method developed to analyze filters. Using cancer cell lines spiked in normal blood and filtered by ISET, we developed a multi-step process where filters were (i) treated by four-color fluorescent staining, (ii) scanned using an Ariol scanning system and automatically analyzed to select CD45^−^ from CD45^+^ cells, (iii) treated by a cytomorphological dye, (iv) scanned and analyzed again using the Ariol scanning system to gather for cells of interest phenotypical and cytomorphological images, and cell characteristics (Fig. 1a). To identify CTCs potentially undergoing EMT, four-color fluorescent staining was carried out using epithelial (pan-cytokeratins, EpCAM), mesenchymal (vimentin or N-cadherin) markers, CD45 and nuclear staining (DAPI or Hoechst 33342).

**Fig. 2:**
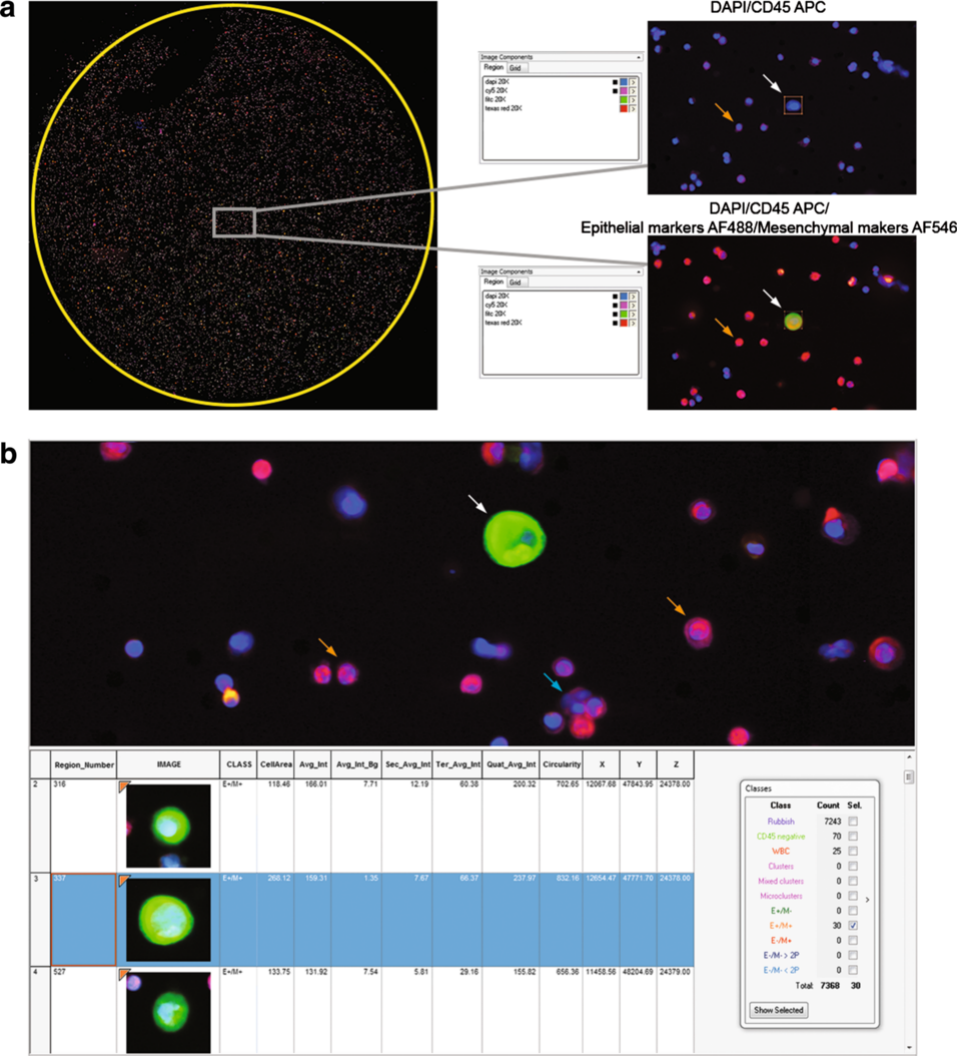
Scanning and image gallery of A459 cells spiked in normal blood and filtered by ISET. **a** Images of the whole ISET filter (left panel). Grey windows and right panels show DAPI/CD45 APC and DAPI/CD45 APC/Epithelial markers AF488/Mesenchymal markers AF546 channels. **b** Zoom in images for DAPI/CD45 APC/Epithelial markers AF488/Mesenchymal markers AF546 channels. The image gallery of A549 cells and cell characteristics are shown. Cell categories after automated selection of CD45^−^ cells and manual review of images are shown. The white arrow indicates an A549 cell, orange arrows indicate CD45^+^ cell into pores, the blue arrow a CD45^+^ cell outside a pore. Abbreviations: Avg_Int, DAPI average intensity; Avg_Int_Bg, background average intensity, Sec_Avg_Int, CD45 average intensity; Ter_Avg_Int, AF488 average intensity; Quat_Avg_Int, AF546 average intensity

Three scanning parameters including gain and exposure time – which operate on fluorochrome intensity – and offset – which operates on the fluorescent background of the membrane – were critical for the automated selection of CD45^−^ cells. CD45 exposure time was systematically adjusted as high as possible to optimally discriminate CD45^−^ and CD45^+^ cells (Fig. 2a). The exposure time of epithelial and mesenchymal markers was adjusted to detect low marker expression as expected in CTCs undergoing EMT (Fig. 2a). Offset calibration in the DAPI channel was critical to fully demarcate nuclei and automatically select individual CD45^−^ from CD45^+^ cells. To adjust for the uneven surface of the membrane, the focus was determined before scanning using nine focus points across the filter, then readjusted every frame during scanning (~350 times). Using this scanning method, the total number of cells (mostly CD45^+^) counted per 1 mL ISET spot was highly variable, ranging from 6000 to 20000. The automated selection of CD45^−^ cells was based on three criteria: (i) intensity of DAPI, (ii) intensity of CD45, (iii) a nuclear area superior to 55 μm^2^ (an area of 50 μm^2^ corresponds to a pore of 8 μm diameter). In order to select clusters, and especially mixed clusters which contain both CTCs and CD45^+^ cells, a nuclear area superior to 150 μm^2^ (equivalent of three pores) was added as a fourth criteria, and the CD45 intensity was not considered in this situation. All selected DAPI^+^/CD45^−^ elements (usually ranging from 10 to 150 elements per 1 mL ISET spot) were reviewed manually and classified according to their phenotype and nucleus size. Eight categories were established including exclusively epithelial cells (E^+^/M^−^), cells in EMT (E^+^/M^+^), mesenchymal cells (E^−^/M^+^), cells with no markers and a nucleus diameter superior to 16 μm (E^−^/M^−^ > 2 pores), cells with no markers and a nucleus diameter inferior to 16 μm (E^−^/M^−^ < 2 pores), CTC clusters (number of CTCs ≥ 4), mixed clusters (number of CD45^+^ cells and CTCs ≥ 4), and microclusters (number of CD45^−^ and/or CTC = 3). The characteristics of each selected element such as cell area and fluorescence intensities were detailed in an image gallery. An example of A549 cancer cells spiked in normal blood and treated as mentioned above is shown in Fig. 2.

**Fig. 3:**
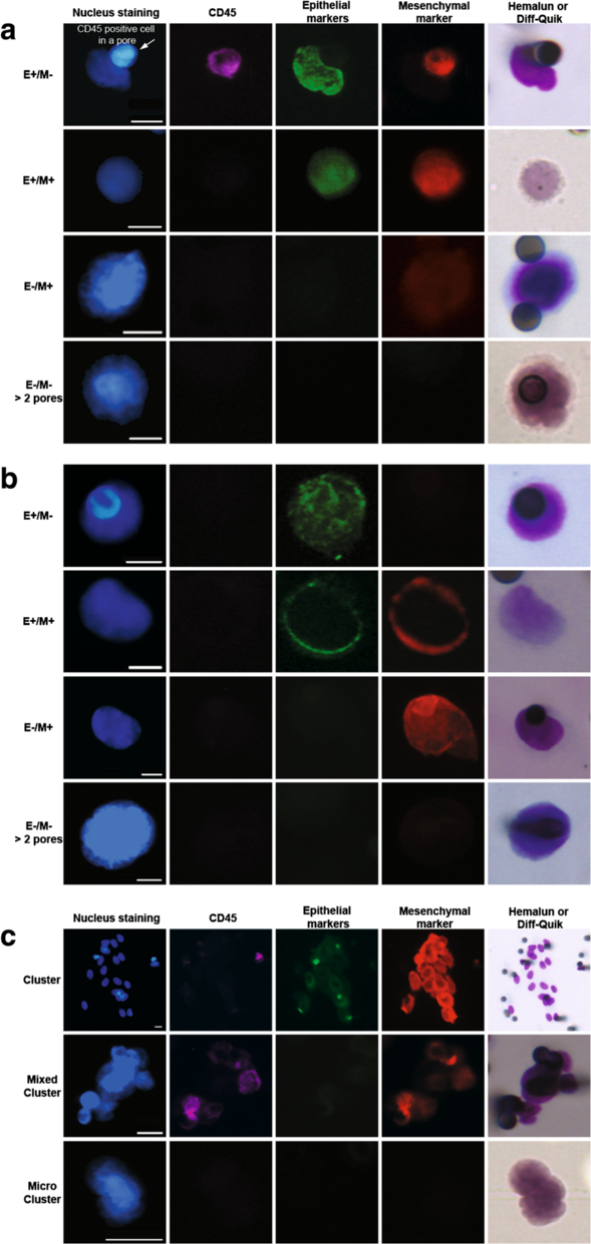
Examples of isolated CTCs (E^+^/M^−^, E^+^/M^+^, E^−^/M^+^, E^−^/M^−^ > 2 pores respectively) identified by combined fluorescent staining and high-resolution cytomorphology in representative **a** NSCLC and **b** mPCa patients. **c** Examples of CTC clusters, mixed clusters and microclusters respectively in NSCLC and/or mPCa patients. Scale: white bars = 10 μm

High-resolution cytomorphology was carried out by staining filters with either Mayer Hemalun or Diff-Quik, and scanning in brightfield. Cytomorphological images were precisely relocated within CD45^−^ cell subpopulations using the Ariol scanning system and validated by an experienced cytopathologist (PV). Five criteria were used to define an epithelial CTC including i) presence of a well-defined and non-damaged nucleus inside the cytoplasm, ii) absence of CD45 expression, iii) expression of epithelial markers, iv) nuclear size superior to 1.5 pore diameter (i.e. 12 μm), v) presence of a well-defined cytoplasm. Examples of isolated CTCs from NSCLC and mPCa patients identified using these five criteria and harboring only epithelial markers or both epithelial and mesenchymal markers are shown in Fig. 3 (a and b). In the absence of epithelial marker expression, CTCs were defined as cells presenting all the following cytomorphological criteria: i) nucleus size superior to two pores (i.e. ≥ 16 μm), ii) nucleus irregularity, iii) high nuclear-to-cytoplasmic ratio. CTCs with no detectable epithelial marker expression but cytomorphological characteristics of CTCs were consistently detected in both NSCLC and mPCa patients as shown in Fig. 3 (a and b). Examples of clusters from NSCLC and/or mPCa patients are shown in Fig. 3c.

### Semi-automated microscopy method to identify filtration-enriched CTCs by combined phenotypical and FA-FISH analysis

We previously reported a method for FA-FISH on ISET filters, optimized to decrease non-specific fluorescent probe binding to the filter membrane and confer high cell recovery [18, 20]. Scanning software is commonly able to analyze around 100 cells per tumor sample by FISH, and it was therefore not possible to scan all cells (6000 to 20,000, as mentioned above) present on a 1 mL ISET spot. To decrease the numbers of cells to scan, we limited the FISH analysis to CD45^−^ cells: this option provided the additional advantage of excluding CD45^+^ subpopulations which could occasionally harbor unspecific FISH rearrangement signals when damaged (data not shown). We established a second multi-step process where filters were (i) treated by fluorescent staining, (ii) scanned on the Ariol scanning system and analyzed automatically to locate CD45^−^ cells as described above, (iii) treated by FA-FISH, (iv) scanned in the small regions containing the CD45^−^ cells, (v) and analyzed for detection, interpretation and validation of FISH signals within CD45^−^ cells (Fig. 1b).

FISH scanning in tumor specimens depends on three parameters: z-stacking (usually ranging from 7 to 11 stacks), the step i.e. the distance between z-stacks (usually 0.6 μm or 0.7 μm), and the exposure time of red and green fluorochromes. FISH scanning of filter enriched-CTCs must take into consideration two particular characteristics of CTCs and filters: first, CTCs are not cut as tissue sections and may have a large nucleus (superior to 30 μm in diameter). These characteristics add to the uneven surface of the filter and strongly influence the above three parameters. Second, CTCs are frequently located in pores which contain DAPI positive debris and fluorescent signals, rendering their analysis challenging.

We first determined the best number of z-stacks to scan filter enriched-CTCs from patients with an *ALK*-rearranged tumor. Six independent scans of the same filters were performed with an increasing number of z-stacks ranging from 5 to 30, at an arbitrary step of 0.6 μm. Thirty was the maximum z-stack number tolerated by the scanner. As shown in Table 1, 30 z-stacks was the optimal setting to scan CTC FISH spots (82 % detection rate) in an patient with *ALK*-rearranged tumor, while fewer spots were detected at lower numbers of z-stacks. Thirty z-stacks were also optimal to scan CTC FISH spots in patients with a *ROS1*-rearranged tumor (91 % detection rate) (Table 2). In a patient with *ERG*-rearranged tumor, 30 stacks also allowed a 95 % detection rate of CTC FISH spots (Table 3). Thirty z-stacks were therefore established as the optimal setting for scanning filter-enriched CTCs.

**Table 1:** FISH spots detected per CTC in a patient with an *ALK*-rearranged tumor depending on the number of z-stacks

CTC ID	Number of FISH spots					
	5 z-stacks	10 z-stacks	15 z-stacks	20 z-stacks	25 z-stacks	30 z-stacks
1	n.i^a^	9	9	13	10	**18**
2	3	8	6	**16**	15	**16**
3	n.i^a^	3	2	8	7	**12**
4	4	11	2	4	13	**13**
5	n.i^a^	5	6	8	8	**17**
6	n.i^a^	12	4	4	2	**16**
7	8	6	8	19	1	**27**
8	2	4	4	8	10	**15**
9	2	2	3	13	7	**16**
10	n.i^a^	22	6	14	17	**27**
11	n.i^a^	4	8	8	10	**16**
12	1	10	6	**16**	12	**16**
13	1	4	2	**16**	2	3
14	n.i^a^	6	9	**14**	**14**	**14**
15	2	4	n.i^a^	4	4	**16**
16	1	10	8	2	7	**16**
17	n.i^a^	6	9	8	**20**	9
18	6	7	7	**16**	14	**16**
19	6	7	n.i^a^	10	12	**18**
20	3	8	6	6	8	**18**
21	1	3	**9**	6	8	8
22	2	5	6	n.i.^a^	**14**	8
%^b^	0 %	0 %	4 %	23 %	18 %	82 %

Abbreviations: *ALK* anaplastic lymphoma kinase gene; CTC circulating tumor cell; FISH fluorescence *in situ* hybridization; n.i. non-interpretable

^a^Number of FISH spots is uncountable due to non-optimal focus in the DAPI channel

^b^Percentage of cases where a higher number of FISH spots were observed

The numbers in bold correspond to the highest number of spots for this CTC

**Table 2:** FISH spots detected per CTC in a patient with a *ROS1*-rearranged tumor depending on the number of z-stacks

CTC ID	Number of FA-FISH spots					
	5 stacks	10 stacks	15 stacks	20 stacks	25 stacks	30 stacks
1	9	n.i^a^	6	14	14	**30**
2	4	**10**	**10**	**10**	**10**	**10**
3	12	22	18	**28**	**28**	**28**
4	3	2	n.i^a^	13	**14**	**14**
5	**4**	**4**	**4**	**4**	**4**	**4**
6	26	28	26	**34**	**34**	**34**
7	6	15	11	20	**28**	**28**
8	36	42	48	52	**62**	**62**
9	10	14	18	20	**30**	**30**
10	13	20	22	26	**30**	**30**
11	2	12	12	13	**14**	**14**
12	6	6	8	10	**14**	**14**
13	4	6	6	8	**14**	**14**
14	8	13	20	**32**	**32**	**32**
15	10	14	14	**16**	**16**	**16**
16	10	12	13	**16**	**16**	**16**
17	2	6	4	15	**16**	**16**
18	4	11	16	**25**	**25**	**25**
19	20	33	26	46	55	64
20	12	14	12	14	15	14
21	11	n.i^a^	20	24	**28**	**28**
22	10	12	12	**20**	19	12
%^b^	4 %	9 %	9 %	41 %	86 %	91 %

Abbreviations: CTC circulating tumor cell; FISH fluorescence *in situ* hybridization; n.i. non-interpretable; *ROS1 c*-*ros oncogene 1*

^a^Number of FISH spots is uncountable due to non-optimal focus in the DAPI channel

^b^Percentage of cases where a higher number of FISH spots were observed

The numbers in bold correspond to the highest number of spots for this CTC

**Table 3:** FISH spots detected per CTC in a patient with an *ERG*-rearranged tumor depending on the number of z-stacks

CTC ID	Number of FA-FISH spots					
	5 z-stacks	10 z-stacks	15 z-stacks	20 z-stacks	25 z-stacks	30 z-stacks
1	5	9	2	10	16	**22**
2	23	32	22	32	33	**36**
3	4	12	6	15	**16**	**16**
4	11	20	10	24	30	**32**
5	10	6	12	**16**	14	**16**
6	26	14	2	30	31	**45**
7	34	4	2	39	**59**	**59**
8	15	4	4	27	**31**	**31**
9	14	8	2	31	33	**34**
10	6	2	16	26	26	**31**
11	2	2	4	15	16	**22**
12	14	32	2	60	58	**61**
13	13	0	20	**28**	**28**	**28**
14	10	10	12	**35**	32	**35**
15	17	10	10	56	53	**65**
16	16	6	8	28	30	**32**
17	6	6	10	14	15	**16**
18	5	14	2	14	14	**20**
19	8	4	6	16	26	**31**
20	30	20	26	**52**	**52**	44
21	4	16	6	12	18	**27**
22	7	n.i^a^	6	**30**	29	**30**
%^b^	0 %	0 %	0 %	23 %	23 %	95 %

Abbreviations: CTC circulating tumor cell; *ERG* v-ets avian erythroblastosis virus E26 oncogene homolog; FISH fluorescence *in situ* hybridization; n.i. non-interpretable

^a^Number of FISH spots is uncountable due to non-optimal focus in the DAPI channel

^b^Percentage of cases where a higher number of FISH spots were observed

The numbers in bold correspond to the highest number of spots for this CTC

We next examined the influence of the step on the number of FISH spots. Scanning of the same filters was performed with an increasing step, ranging from 0.5 μm to 0.8 μm. As shown in a patient with *ALK*-rearranged tumor (Table 4), a maximal number of FISH spots was detected at a step of 0.6 μm (92 % detection rate). This was confirmed in filters from patients with *ROS1*- and *ERG*-rearranged tumors (Additional file 2: Table S1 and Additional file 3: Table S2).

**Table 4:** FISH spots detected per CTC in a patient with an *ALK*-rearranged tumor depending on the step

CTC ID	Number of FA-FISH spots			
	0.5 μm	0.6 μm	0.7 μm	0.8 μm
1	**16**	**16**	**16**	**16**
2	**16**	**16**	**16**	**16**
3	**32**	**32**	**32**	28
4	**32**	**32**	28	27
5	**16**	**16**	14	13
6	**32**	**32**	**32**	**32**
7	**14**	**14**	**14**	**14**
8	13	**16**	12	16
9	29	28	**32**	**32**
10	**16**	**16**	**16**	**16**
11	**16**	**16**	**16**	**16**
12	28	26	**31**	**30**
13	**32**	**32**	**32**	30
14	**32**	**32**	**32**	**32**
15	**14**	**14**	**14**	**14**
16	27	**28**	18	27
17	**16**	**16**	**16**	**16**
18	**14**	**14**	**14**	12
19	30	**32**	30	27
20	**16**	**16**	**16**	14
21	**16**	**16**	**16**	**16**
22	**32**	**32**	31	22
23	**32**	**32**	**32**	27
24	**32**	**32**	**32**	**32**
%^a^	79 %	92 %	75 %	58 %

Abbreviations: *ALK* anaplastic lymphoma kinase gene; CTC, circulating tumor cell; FISH, fluorescence *in situ* hybridization

^a^Percentage of cases where a higher number of FISH spots were observed

The numbers in bold correspond to the highest number of spots for this CTC

**Fig. 4:**
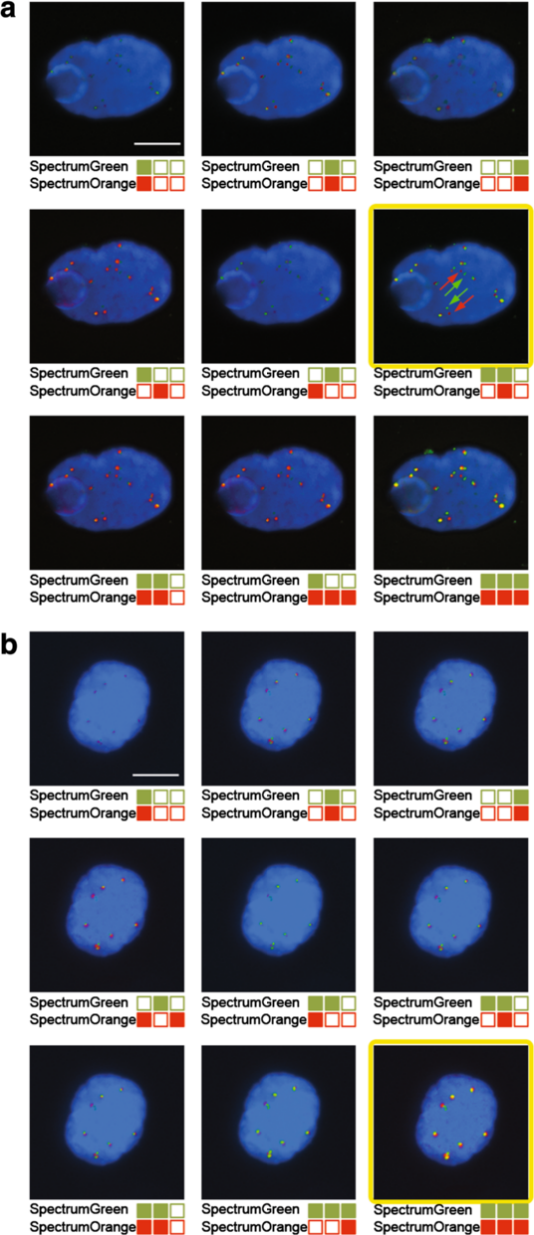
Detection of *ALK*-rearrangement and gain of *ALK*-native copies in CTCs from a patient with an *ALK*-rearranged tumor using the multi-exposure protocol. *ALK*-gene status was tested in filtration enriched-CTCs by combined fluorescent staining and fluorescent *in situ* hybridization (FISH). FISH signals were captured with the multi-exposure protocol and 9 out of the 27 possibilities are shown. Images of best exposure level for red and green signals are framed in yellow. **a** Detection *ALK*-rearrangement. Green and red arrows indicate the break apart signal. **b** Detection of gain of *ALK*-native copies. Scale: white bars = 10 μm

**Fig. 5:**
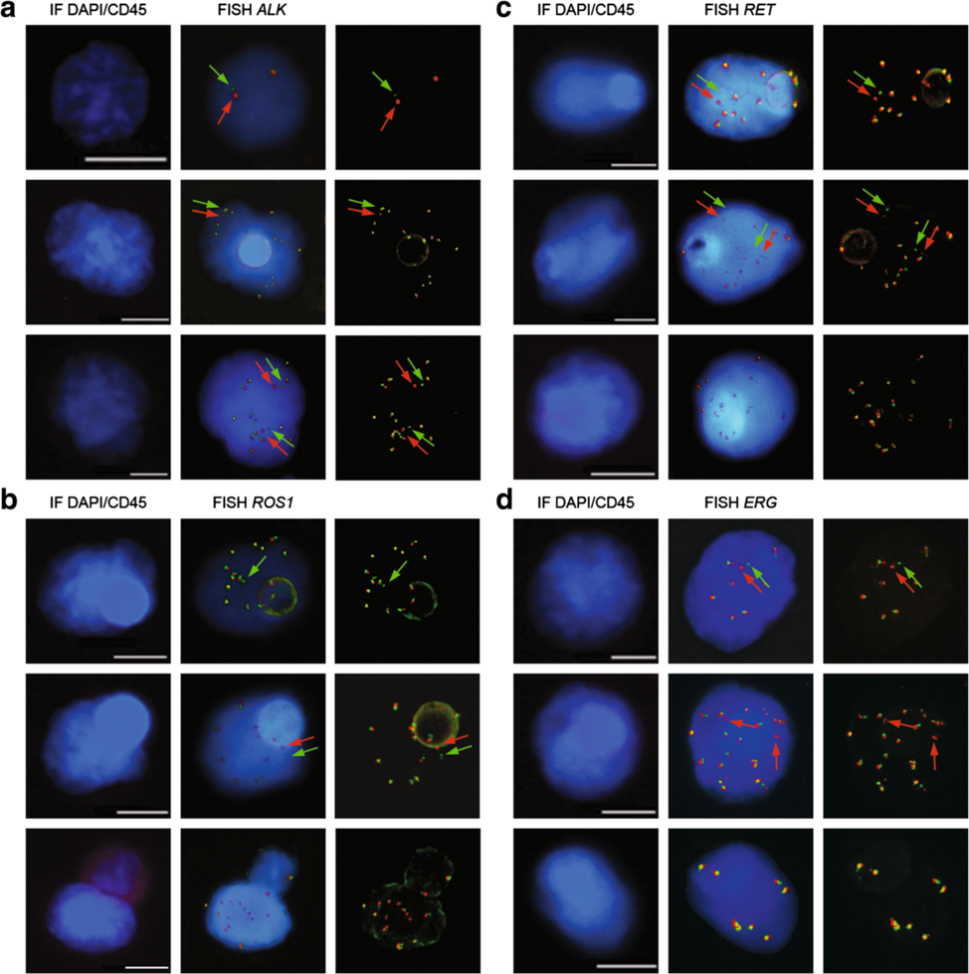
Examples of gene-rearrangement detection in filtration enriched-CTCs from NSCLC and mPCa patients by combined fluorescent staining and fluorescent *in situ* hybridization (FISH). **a** Example of *ALK*-rearrangement detection in NSCLC patients with an *ALK*-rearranged tumor. **b** Example of *ROS1*-rearrangement detection in NSCLC patients with a *ROS1*-rearranged tumor. **c** Example of *RET*-rearrangement detection in NSCLC patients with a *RET*-rearranged tumor. **d** Example of *ERG*-rearrangement detection in mPCa patients with an *ERG*-rearranged tumor. Gene rearrangements are shown by green and red. Scale: white bars = 10 μm

The exposure time of red and green signals was observed to strongly influence the intensity, size and thickness of FISH spots. Because FISH signal intensities were highly variable from one ISET filter region to another one, we developed a multi-exposure protocol to analyze FISH signals consisting of three exposure times for both the green and red fluorochromes (Fig. 4). Twenty-seven settings of exposure time were in theory possible allowing for each candidate CTC the selection of the best setting. As shown in Fig. 4a, the highest exposure was not systematically the best setting, as thick signals can give a false idea of the distance between two spots. To identify CTCs harboring abnormal FISH patterns, signals present in DAPI^+^/CD45^−^ cells were systematically validated by an experienced cytogeneticist (NA). Examples of filtration-enriched CTCs harboring *ALK*-, *ROS1*-, *RET*-, or *ERG*-gene alterations from NSCLC and mPCa are illustrated in Fig. 5.

## Discussion

Here we reported the development of a semi-automated microscopy method for analyzing filtration-enriched CTCs using two combined assays. The first assay allowed identification of CTCs by combining four-color fluorescence to high-resolution cytomorphological analysis. The microscopy method of this first assay included establishment of scanning parameters, definition of criteria for creating an image gallery of CD45^−^ cells, and definition of criteria to identify CTCs among CD45^−^ cells based on both phenotypical and true cytomorphological characteristics. The second process was developed to identify molecular biomarkers in CTCs by combining fluorescent staining and FISH. After identification and location of CD45^−^ cells on filters as established in the first assay, the method included the establishment of parameters for scanning FA-FISH into filter regions containing CTC candidates, and the analysis of FISH using a multi-exposure protocol for optimal definition of signals. These two assays were possible due to the capacity of the Ariol scanning system to perfectly relocate images i.e. images of fluorescence and cytomorphology, and images of fluorescence and FA-FISH signals in CD45^−^ cells.

In contrast to antibody-based immunomagnetic enrichment, high numbers of CD45^+^ cells are retained by most filtration systems [10, 26, 32, 37]. The automated selection of CD45^−^ cells greatly reduces the number of cells to analyze (here approximately 100 cells instead of 6000 to 20000) thereby improving the feasibility of the assay and the reliability of the results. CTCs were identified among selected CD45^−^ cell candidates by examining high-resolution images from both four-color fluorescence and cytomorphology. High-resolution cytomorphology is only possible when cells collected on filters are intact and their morphology well preserved during the filtration process. Although very time-consuming given the number of spots to analyze and the high number of CD45^+^ cells retained per spot, ISET offers the advantage of preserving cellular integrity. In both NSCLC and mPCa patients, we observed CTCs that were negative for epithelial and mesenchymal markers but were validated as true CTCs by a cytopathologist (PV). One cannot completely exclude that CTCs undergoing EMT and expressing a low level of epithelial and mesenchymal markers may be missed by our immunofluorescent assay. Furthermore as shown here, the level of both epithelial and/or mesenchymal marker expression detected in actual filtration-enriched CTCs can be substantially lower than that of spiked cell lines, a difference we also observed using manual microscopy. Although we have no evident explanation for this, the combination of four-color fluorescence to high-resolution cytomorphology contributes to a better characterization of such CTCs and reliability of the results. Therefore by combining phenotypic and cytomorphological data and high-resolution images, the approach described here enables us to better characterize CTCs as well as detect more CTCs than with phenotypic analyses alone, further highlighting the considerable phenotypic and cytomorphological heterogeneity of CTCs.

FISH analysis of tumor specimens for diagnostic testing of biomarkers such as *ALK*- or *ROS1*-rearrangement is in most cases still done manually by cytogeneticists. Tumor cells are identifiable by their morphology and the manual analysis offers the possibility to perform as many stacks as necessary for capturing all FISH spots. Manual FISH analysis of filters is laborious given the number of CD45^+^ cells retained per filter. It may also be a source of errors since it is well known by cytogeneticist that apoptotic CD45^+^ cells may harbor non-specific break apart FISH signals. Given the size of CTCs and the absence of an even filter surface, 30 z-stacks spaced 0.6 μm apart were required to capture a maximum of FISH spots and identify *ALK*-, *ROS1*- and *ERG*-rearrangement in CTCs from NSCLC and mPCa patients. Pre-analytical steps during ISET filtration were found critical for the quality of FA-FISH and optimal scanning, especially the possibility to filter at a very low pressure enabling preservation of cell integrity. Unlike tissue sections, standard scanning settings were unable to capture all FISH spots present in filtration-enriched CTCs. The multi-exposure protocol offering twenty-seven exposure settings was therefore essential for optimal capture and definition of FISH signals that were systematically validated by an experienced cytogeneticist (NA).

Multiple studies have highlighted the important phenotypic and genetic heterogeneity of CTCs which cannot be covered by a single CTC isolation technique. Some of these studies have also shown the capacity of filtration to enrich CTCs harboring distinct epithelial-to-mesenchymal phenotypes, capturing higher numbers of CTCs compared to antibody-based methods, mostly in NSCLC and mPCa. As a consequence of these advantages and in particular because the number of CTCs is critical for assessing molecular biomarkers, filtration based enrichment methods may be a preferred technique to progress CTCs as a non-invasive approach for selecting specific therapies. Given this clinical perspective, automation of microscopy analysis is a necessary step to improve reproducibility of analyses, reduce risks of errors, inter-operator differences, and progress towards a standardization of filtration-enriched CTC analysis. Furthermore, a second advantage of the automated analysis of filtration-enriched CTC is the possibility to combine successive assays on the same filter. Automation allows precise location of cells on filters, thus relocating cell data and images from successive experiments performed on the same filter. The combination of assays increases the amount of available information, contributing to a better characterization of CTCs and reliability of the results.

## Conclusions

While offering important advantages such as sensitivity of CTC capture and flexibility for CTC characterization, filtration systems have disadvantages that make the automation of microscopy challenging to implement. Manual analysis of filtration-enriched CTCs is time-consuming and highly operator-dependent for large scale analyzes and clinical validation of CTC filtration assays. Here we report a semi-automated microscopy method established to identify filtration-enriched CTCs from NSCLC and mPCa, and detect molecular biomarkers such as *ALK*-, *ROS1*- and *ERG*-rearrangements in CTCs. This method may help to improve reproducibility, reduce inter-operator difference and duration of filtration-enriched CTC assays. In addition, data issued from successive experiments may be gathered, further improving and refining CTC characterization. By increasing the feasibility and reliability of filtration-enriched CTC assays, the present method may be helpful to progress towards their standardization and validation. This will contribute to advancing these assays towards the clinic using a routine clinical test.

## Abbreviations

AF, alexa fluor; *ALK*, anaplastic lymphoma kinase; APC, allophycocyanin; *AR*, androgen receptor; CK, cytokeratin; *c*-*MET*, MET proto-oncogene; CTC, circulating tumor cell; Cy5, cyanine 5; DAPI, 4′,6-diamidino-2-phenylindole; DMEM, dulbecco/vogt modified eagle’s minimal essential medium; EDTA, ethylenediaminetetraacetic acid; *EGFR*, epidermal growth factor receptor; EMT, epithelial-mesenchymal transition; EpCAM, epithelial cell adhesion molecule; *ERG*, v-ets avian erythroblastosis virus E26 oncogene homolog; FA-FISH, filter-adapted-FISH; FDA, U.S. Food and Drug Administration; FGFR1, fibroblast growth factor receptor 1; *FIP1L1*, FIP1 like 1; FISH, fluorescence *in situ* hybridization; FITC, fluorescein isothiocyanate; HCL, hydrogen chloride; IgG, immunoglobulin G; ISET, isolation by size of epithelial tumor cells; MB, multibay; mPCa, metastatic prostate cancer; NSCLC, non-small-cell lung cancer; PBS, phosphate buffered saline; *PDGFRA*, platelet-derived growth factor receptor, alpha polypeptide; *RET*, RET proto-oncogene; *ROS1*, c-ros oncogene 1; RPMI, roswell park memorial institute medium; TBS, tris- buffered saline

## Additional files

Additional file 1: Figure S1. Examples of gene rearrangement and gain/amplification detection in filtration enriched-cell lines by filter-adapted-FISH (FA-FISH). (A) Example of gene rearrangement detection. (B) Example of gain/amplification detection. Scale: white bars = 10 μm. (TIF 8523 kb) Additional file 2: Table S1. FISH spots detected per CTC in a patient with a *ROS1*-rearranged tumor depending on the step. (DOC 68 kb) Additional file 3: Table S2. FISH spots per CTC of a patient with an *ERG*-rearranged tumor depending on the step. (DOC 63 kb)
