# Supplementary figures and images for: Method for semi-automated microscopy of filtration-enriched circulating tumor cells
Source: BMC Cancer. 2016 Jul 14;16:477. doi: 10.1186/s12885-016-2461-4 (PMC4946105; doi:10.1186/s12885-016-2461-4)

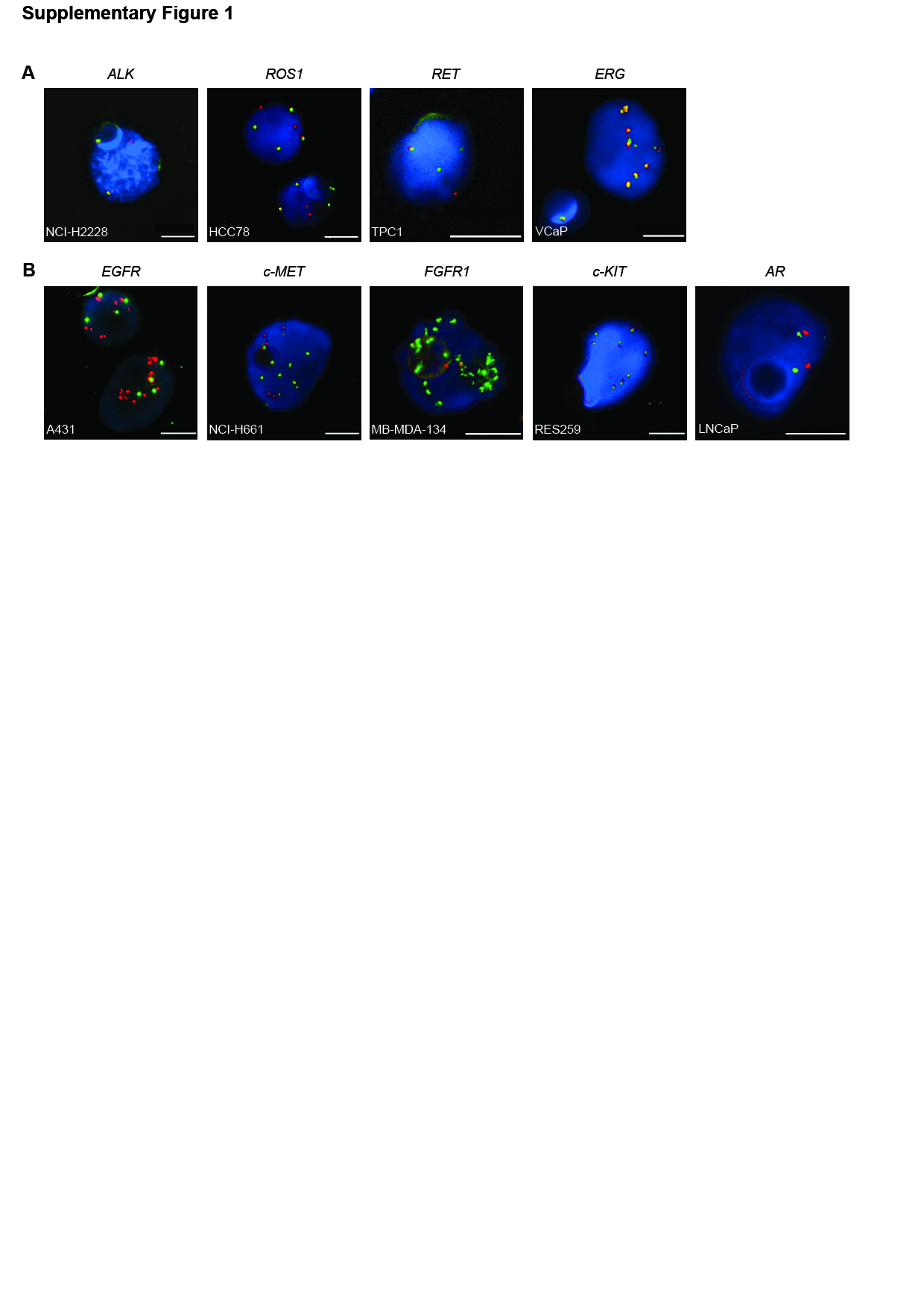

Supplement: Additional file 1: Figure S1. — Examples of gene rearrangement and gain/amplification detection in filtration enriched-cell lines by filter-adapted-FISH (FA-FISH). (A) Example of gene rearrangement detection. (B) Example of gain/amplification detection. Scale: white bars = 10 μm. (TIF 8523 kb) [file 12885_2016_2461_MOESM1_ESM.tif]
