# Supplementary material for: Method for semi-automated microscopy of filtration-enriched circulating tumor cells
Source: BMC Cancer. 2016 Jul 14;16:477. doi: 10.1186/s12885-016-2461-4 (PMC4946105; doi:10.1186/s12885-016-2461-4)
Supplement: Additional file 2: Table S1. — FISH spots detected per CTC in a patient with a ROS1-rearranged tumor depending on the step. (DOC 68 kb) [file 12885_2016_2461_MOESM2_ESM.doc]

**Supplementary Table 1.** FISH spots detected per CTC in a patient with a *ROS1*-rearranged tumor depending on the step.

| **CTC ID** | **Number of FA-FISH spots** | | | | | | | |
| --- | --- | --- | --- | --- | --- | --- | --- | --- |
|  |  | **0.5 µm** |  | **0.6 µm** |  | **0.7 µm** |  | **0.8 µm** |
| 1 |  | 11 |  | **30** |  | n.i* |  | 28 |
| 2 |  | 8 |  | 16 |  | 17 |  | **26** |
| 3 |  | **10** |  | **10** |  | **10** |  | **10** |
| 4 |  | 29 |  | 28 |  | **30** |  | **30** |
| 5 |  | n.i* |  | **4** |  | **4** |  | **4** |
| 6 |  | 30 |  | **34** |  | **34** |  | **34** |
| 7 |  | 26 |  | **28** |  | **28** |  | 26 |
| 8 |  | 27 |  | **28** |  | **28** |  | 22 |
| 9 |  | 53 |  | 62 |  | 61 |  | **63** |
| 10 |  | 29 |  | **30** |  | 29 |  | 28 |
| 11 |  | 19 |  | **32** |  | n.i* |  | **32** |
| 12 |  | **6** |  | **6** |  | **6** |  | n.i* |
| 13 |  | **30** |  | **30** |  | **30** |  | **30** |
| 14 |  | **14** |  | **14** |  | **14** |  | 12 |
| 15 |  | **14** |  | 13 |  | **14** |  | 12 |
| 16 |  | 10 |  | **14** |  | **14** |  | n.i* |
| 17 |  | **32** |  | **32** |  | 31 |  | 31 |
| 18 |  | **16** |  | **16** |  | n.i* |  | **16** |
| 19 |  | **16** |  | **16** |  | 13 |  | 12 |
| 20 |  | **16** |  | **16** |  | 15 |  | 15 |
| 21 |  | 12 |  | **16** |  | n.i* |  | 10 |
| 22 |  | 25 |  | 25 |  | **27** |  | 26 |
| 23 |  | 8 |  | **14** |  | **14** |  | **14** |
| 24 |  | 14 |  | **15** |  | 13 |  | 14 |
| 25 |  | 15 |  | **28** |  | n.i* |  | 18 |
| 26 |  | 12 |  | 12 |  | **13** |  | **13** |
| **%**** |  | **35%** |  | **77%** |  | **54%** |  | **42%** |

Abbreviations: CTC, circulating tumor cell; FISH, fluorescence *in Situ* hybridization ; n.i., non-interpretable; *ROS1*, *c-ros oncogene 1*.

* Number of FISH spots is uncountable due to non-optimal focus in the DAPI channel.

** Percentage of cases where a higher number of FISH spots were observed.

The numbers in bold orange correspond to the highest number of spots for this CTC.
