# Supplementary material for: Method for semi-automated microscopy of filtration-enriched circulating tumor cells
Source: BMC Cancer. 2016 Jul 14;16:477. doi: 10.1186/s12885-016-2461-4 (PMC4946105; doi:10.1186/s12885-016-2461-4)
Supplement: Additional file 3: Table S2. — FISH spots per CTC of a patient with an ERG-rearranged tumor depending on the step. (DOC 63 kb) [file 12885_2016_2461_MOESM3_ESM.doc]

**Supplementary Table 2.** FISH spots per CTC of a patient with an *ERG*-rearranged tumor depending on the step.

| **CTC ID** | **Number of FA-FISH spots** | | | | | | | |
| --- | --- | --- | --- | --- | --- | --- | --- | --- |
|  |  | **0.5 µm** |  | **0.6 µm** |  | **0.7 µm** |  | **0.8 µm** |
| 1 |  | 28 |  | **31** |  | 27 |  | 20 |
| 2 |  | 26 |  | **28** |  | **28** |  | 27 |
| 3 |  | 32 |  | **35** |  | 28 |  | 25 |
| 4 |  | 50 |  | **65** |  | 53 |  | n.i* |
| 5 |  | 24 |  | **26** |  | 21 |  | 12 |
| 6 |  | **23** |  | **23** |  | 21 |  | n.i. |
| 7 |  | 12 |  | **19** |  | 13 |  | 17 |
| 8 |  | **32** |  | **32** |  | 30 |  | 30 |
| 9 |  | 17 |  | 16 |  | **20** |  | 18 |
| 10 |  | **52** |  | **52** |  | 46 |  | 28 |
| 11 |  | 27 |  | **39** |  | 28 |  | n.i* |
| 12 |  | 22 |  | **27** |  | 22 |  | 22 |
| 13 |  | 28 |  | **34** |  | 25 |  | 21 |
| 14 |  | 35 |  | **38** |  | 25 |  | 35 |
| 15 |  | 32 |  | 50 |  | 55 |  | **62** |
| 16 |  | 19 |  | **23** |  | 19 |  | 16 |
| 17 |  | 13 |  | **16** |  | 15 |  | 11 |
| 18 |  | 30 |  | 24 |  | 32 |  | **34** |
| 19 |  | 30 |  | **32** |  | 30 |  | 24 |
| 20 |  | 18 |  | **30** |  | 24 |  | 26 |
| 21 |  | 14 |  | **20** |  | 11 |  | 15 |
| 22 |  | 10 |  | 8 |  | 8 |  | **12** |
| **%**** |  | **14%** |  | **82%** |  | **9%** |  | **14%** |

Abbreviations: CTC, circulating tumor cell; *ERG*, v-ets avian erythroblastosis virus E26 oncogene homolog; FISH, fluorescence *in situ* hybridization; n.i., non-interpretable.

* Number of FISH spots is uncountable due to non-optimal focus in the DAPI channel.

** Percentage of cases where a higher number of FISH spots were observed.

The numbers in bold orange correspond to the highest number of spots for this CTC.
